# Expanding the Trifluoroborate Toolbox: Pyridinium Scaffolds for PET Tracer Development

**DOI:** 10.1021/acscentsci.6c00966

**Published:** 2026-06-16

**Authors:** Antonio A. W. L. Wong, David M. Perrin

**Affiliations:** † Department of Basic and Translational Research, BC Cancer Research Institute, Vancouver, BC V5Z 1L3, Canada; ‡ Department of Chemistry, 8166 University of British Columbia, Vancouver, BC V6T 1Z1, Canada

## Abstract

One-step synthesis and tunable physicochemical properties position pyridinium-trifluoroborates as a versatile complement to existing radioprosthetics.

## Graphical abstract

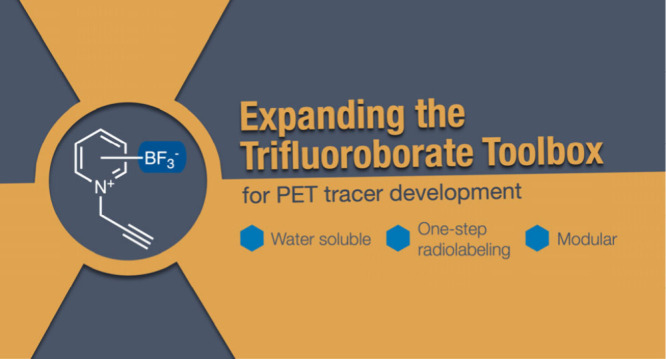

In a recent issue of *ACS Central Science*, Zhang, Fang, Ma, and co-workers introduce a library of pyridinium trifluoroborate (PyrBF_3_) reagents that expand the toolbox for preparing fluorine-18 labeled tracers used in positron emission tomography (PET).^1^ Diagnostic PET imaging with fluorine-18 occupies a central place in modern nuclear medicine as it enables the noninvasive visualization of biological processes with exquisite sensitivity. However, traditionally, the radiosynthesis of ^18^F-radiotracers remains technically demanding, often requiring multiple steps owing to the well-known low reactivity of solvated fluoride anions. Challenges include a half-life of 109.7 min, the aqueous nature of cyclotron-produced anionic [^18^F]­fluoride that often requires drying to enhance its chemical reactivity, and the need for rapid, high-yielding chemistry under mild conditions.

Zhang, Fang, Ma, and co-workers introduce a library of pyridinium trifluoroborate (PyrBF_3_) reagents that expand the toolbox for preparing fluorine-18 labeled tracers used in positron emission tomography.

Prior to 2008, the kinetic stability of organotrifluoroborates in aqueous conditions was poorly understood. In 2008, Perrin and co-workers first characterized (hetero)­aryltrifluoroborates (**1a**–**b**, Table 1) in terms of their solvolysis rates (*t*_1/2_ on the order of days) with accompanying studies in radiolabeling.^2–5^ Later, strategic placement of a cation near the BF_3_ group afforded ammoniomethyltrifluoroborates (AMBF_3_, **3a**–**b**)^6^ and *N*-propargylpyridinium trifluoroborate (**2d**), exhibiting extended *t*_1/2_ ≈ 15–30 d in phosphate-buffered saline.^7^ Similar strategies employing *ortho*-phosphonium-substituted aryl-BF_3_s (**4**) and *N*-heterocyclic carbene-BF_3_s (NHC-BF_3_, **5**) followed and demonstrated the utility of zwitterionic organotrifluoroborates as a radioprosthetic group^8,9^ that enable one-step/last-step radiofluorination for PET.

**Table 1:**
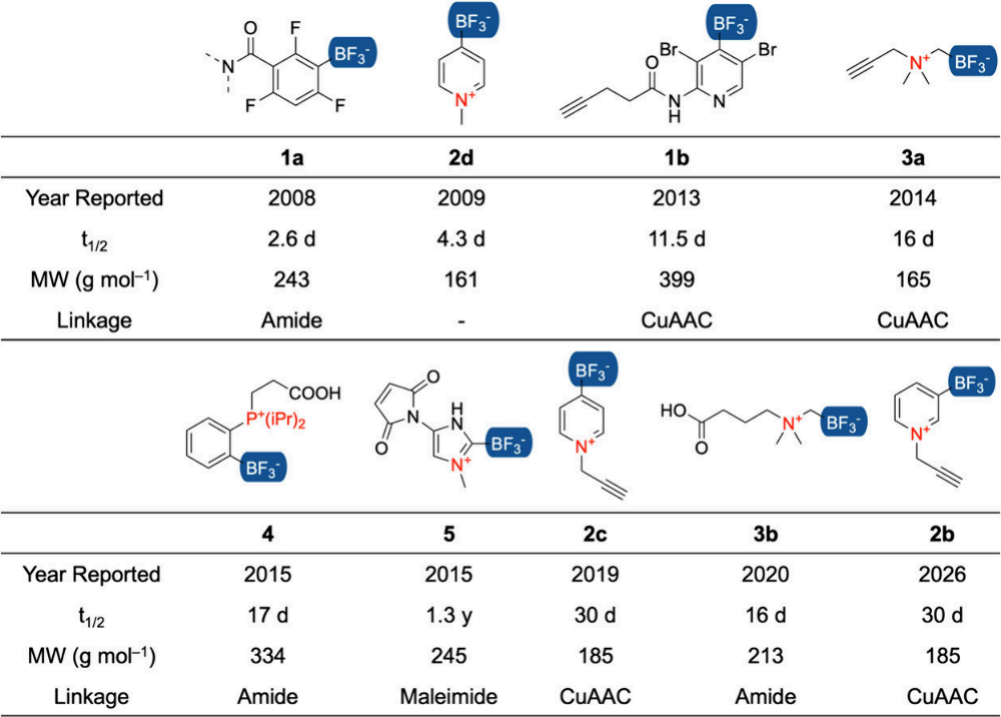
Comparison of Organotrifluoroborate Platforms for PET Tracer Development^a^

a Structures **1**–**5** represent key trifluoroborate scaffolds: (hetero)­aryltrifluoroborates (**1a**–**b**), pyridinium-trifluoroborates (PyrBF_3_, **2a**, **2c**–**d**), ammoniomethyltrifluoroborate (AMBF_3_, **3a**–**b**), phosphonium-trifluoroborate (**4**), and *N*-heterocyclic carbene-trifluoroborate (NHC-BF_3_, **5**). Half-lives (*t*_1/2_) in phosphate-buffered saline at physiological pH. Molecular weights and conjugation chemistries highlight the structural diversity available for tracer optimization.

Li et al. introduce a library of pyridinium trifluoroborates (PyrBF_3_, **2a**–**c**, Figure 1, with more examples available in the manuscript), which are designed for synthetic accessibility and structural diversity.^1^ Building on their previous development of zwitterionic PyrBF_3_ reagents for cross-coupling chemistry,^10^ the authors appreciated that N-alkylation of such *N*-heterocycles **6** could provide conjugation-ready prosthetic groups, reported similarly by Perrin and co-workers.^7^ Treatment of pyridinium trifluoroborates with alkyl halides under basic conditions yields *N*-alkyl PyrBF_3_ reagents **2a**–**c** in a single step. In terms of hydrophilicity of these compounds, log D_7.4_ values span from below −1 to above +1 depending on substitution pattern, offering options for optimizing tracer pharmacokinetics. X-ray crystallography confirms the structural assignments, and aqueous stability studies reveal half-lives ranging from 14 d to 1 y in PBS.

**Figure 1:**
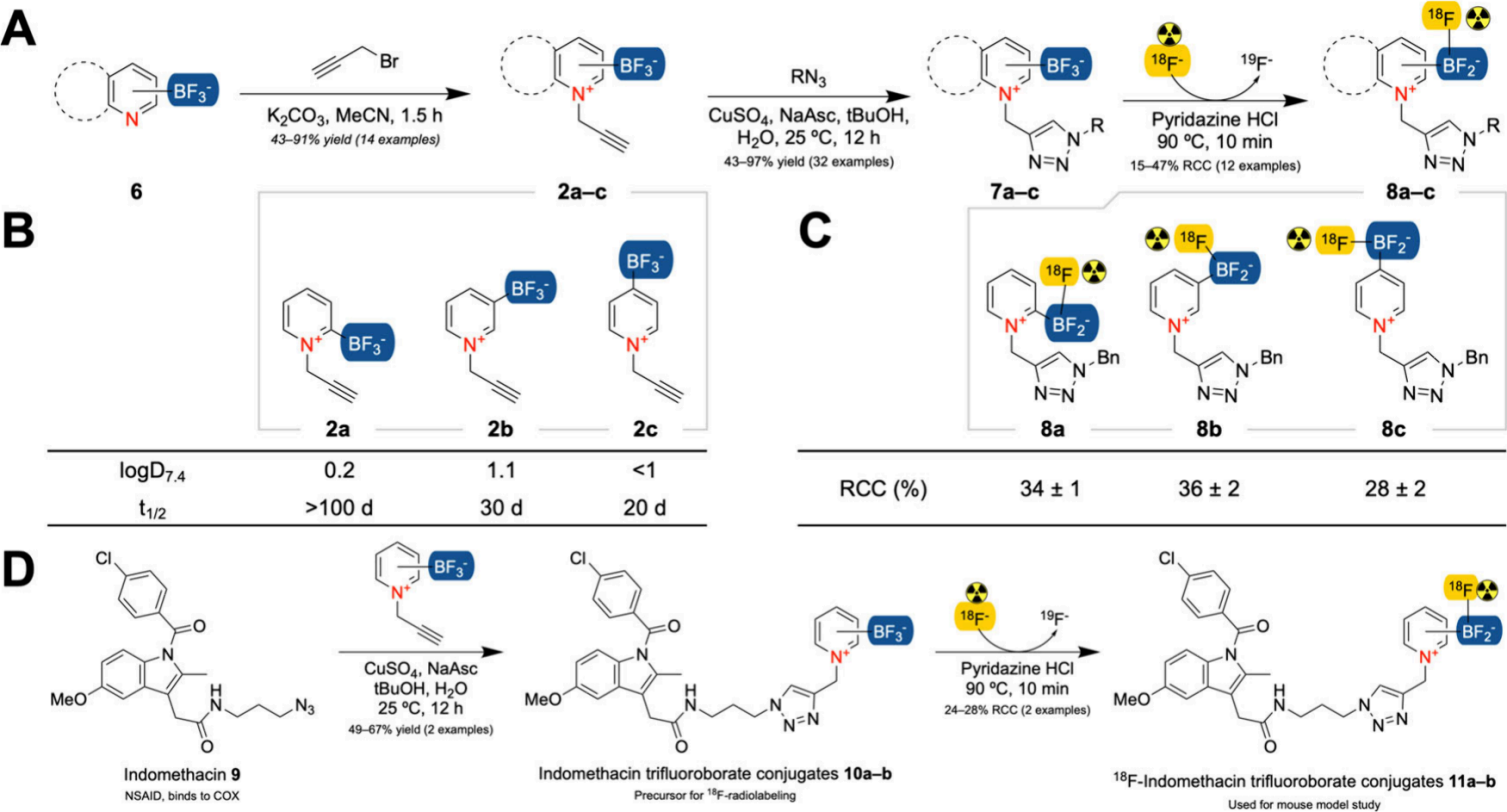
Synthetic route and characterization of pyridinium-trifluoroborate reagents. (A) One-step synthesis of *N*-alkyl PyrBF_3_ reagents (**2a**–**c** and **7a**–**c**) from *N*-heterocycles **6** via alkylation and subsequent copper-catalyzed azide–alkyne cycloaddition (CuAAC) or direct ^18^F-labeling. (B) Physicochemical properties of *N*-alkylated PyrBF_3_ scaffolds showing tunable lipophilicity (log D_7.4_) and aqueous solvolytic stability (*t*_1/2_ in PBS). (C) Radiochemical conversion (RCC) for ^18^F-labeling of PyrBF_3_ conjugates **8a**–**c**. (D) Synthesis and radiolabeling of indomethacin-PyrBF_3_ conjugates **10a**–**b** via CuAAC conjugation followed by ^18^F–^19^F isotope exchange under aqueous conditions.

Building on their previous development of zwitterionic PyrBF_3_ reagents for cross-coupling chemistry, the authors appreciated that N-alkylation of such N-heterocycles could provide conjugation-ready prosthetic groups.

The authors employ copper-catalyzed azide–alkyne cycloaddition (CuAAC) to install the PyrBF_3_ unit onto drug scaffolds, yielding bioconjugates **7a**–**c**. This modular click chemistry approach accommodates diverse functional groups and enables straightforward conjugation. The authors showcase this versatility by preparing PyrBF_3_ conjugates of sulfadoxine, indomethacin **9**, aminoglutethimide, linezolid, and other pharmaceutically relevant molecules in aqueous conditions that leave the trifluoroborate intact. Preliminary ^18^F-radiosyntheses (**8a**–**c**, **11a**–**b**) and microPET/MR imaging in mice demonstrate that indomethacin-PyrBF_3_ conjugates **11a**–**b** undergo efficient isotope exchange and exhibit good *in vivo* stability, with no detectable bone uptake at 2 h postinjection.

This tunability in kinetic stability reflects the principle of microscopic reversibility in BF_3_-based radiochemistry: highly stable organotrifluoroborates that resist solvolytic defluorination are also slow to undergo ^18^F–^19^F isotope exchange, often resulting in lower radiochemical yields. By this corollary, organotrifluoroborates that undergo facile labeling but may also release [^18^F]­fluoride too quickly *in vivo* or upon standing once formulated in PBS. Here, Li et al. demonstrate that C3- (**11a**) and C4-substituted (**11b**) indomethacin conjugates achieve 20–40% radiochemical conversion in 10 min at 90 °C. We suspect that the C2 variant was too stable to be radiofluorinated in useful yields. This structure-dependent reactivity suggests opportunities for optimization within a “Goldilocks zone” where stability and labeling efficiency are appropriately balanced.

Where does this work fit in the broader landscape of radiopharmaceutical chemistry? Water-stable, charge-stabilized trifluoroborates are established tools. AMBF_3_ (**3a**–**b**), phosphonium-BF_3_ (**4**), and NHC-BF_3_ (**5**) platforms have demonstrated preclinical potential. The PyrBF_3_ system contributes synthetic accessibility and structural modularity. Reducing radiosynthetic steps matters when conjugating to complex targeting molecules where late-stage yields are critical. The PyrBF_3_ platform provides chemical diversity: more structural handles, more options for tuning lipophilicity and clearance, and different steric profiles for binding pocket optimization.

The PyrBF_3_ platform provides chemical diversity: more structural handles, more options for tuning lipophilicity and clearance, and different steric profiles for binding pocket optimization.

The authors acknowledge that the pyridinium scaffold introduces a relatively bulky modification compared to other ^18^F-prosthetic groups. This concern certainly merits consideration for small-molecule tracers targeting well-defined binding sites, where steric bulk could disrupt critical interactions. For larger biomolecules or targets with accommodating binding pockets, the PyrBF_3_ group is likely to be well-tolerated.

The critical validation will come from head-to-head comparisons. Can PyrBF_3_-labeled tracers provide superior imaging performance (higher target uptake, higher signal-to-background ratios, or improved pharmacokinetics) compared to AMBF_3_ or other established prosthetics for the same biological target? Certainly, there has been precedence for this approach in the context of a PMSA-targeting tracer for prostate cancer.^7^ Nevertheless, the indomethacin proof-of-concept is encouraging. However, its target cyclooxygenases (COX) 1 and 2 are not clinically validated imaging targets. Demonstrating advantages in tracers for validated targets (integrin receptors, PSMA, fibroblast activation protein) would strengthen the case for adoption.

Beyond technical performance, the operational simplicity of PyrBF_3_ chemistry has broader implications. Requiring fewer synthetic steps and using aqueous click reactions makes this platform accessible to most medicinal chemistry laboratories. Scaling up of these precursors and handles in a process mass intensity (PMI)-conscious manner is a logical next step for translation from a manufacturing standpoint. As precision medicine pushes toward imaging biomarkers for rare diseases and underserved patient populations, the ability to rapidly generate diverse tracer candidates becomes valuable. Streamlined synthesis also has implications for regulatory pathways and manufacturing.

As precision medicine pushes toward imaging biomarkers for rare diseases and underserved patient populations, the ability to rapidly generate diverse tracer candidates becomes valuable. Streamlined synthesis also has implications for regulatory pathways and manufacturing.

The modular nature of the PyrBF_3_ platform aligns with trends toward open-source radiochemistry and collaborative tracer development. Notionally, a one-step radiofluorination and compatibility with standard click chemistry could facilitate knowledge sharing and technology transfer between institutions. In a field where isotope accessibility, manufacturing complexity, and intellectual property can limit availability of imaging agents, platforms that emphasize simplicity and modularity serve public health needs.

The PyrBF_3_ platform expands the repertoire of tools available to radiopharmaceutical scientists while providing structural diversity and tunable stability. Whether or not it becomes a primary choice or a specialized option for certain applications, the field necessarily benefits from having more arrows in the quiver. As radiopharmaceutical development moves toward precision medicine, platforms that offer rapid, modular bioconjugation with diverse physicochemical properties will prove their worth by complementing existing methods and making advanced imaging more accessible to researchers, healthcare practitioners, and patients worldwide.
